# Implications of Human Chorionic Gonadotropin (hCG) Therapy in a Varicocele Patient and Its Effect on In Vitro Fertilization (IVF) Outcome

**DOI:** 10.7759/cureus.54893

**Published:** 2024-02-25

**Authors:** Shradha M Ulhe, Dipali More, Mayur Wanjari

**Affiliations:** 1 Clinical Embryology, Datta Meghe Institute of Higher Education & Research, Wardha, IND; 2 Research and Development, Jawaharlal Nehru Medical College, Datta Meghe Institute of Higher Education & Research, Wardha, IND

**Keywords:** varicocele, fsh, tesa, sperm, infertility

## Abstract

This case study pertains to a 32-year-old male and a 29-year-old female who sought treatment at a fertility clinic due to their primary infertility, which had persisted for over four years. Both individuals underwent comprehensive physical and hormonal examinations; while all reports for the female partner indicated normal findings, the test reports for the male partner revealed the presence of azoospermia and varicocele. The standard surgical protocol for varicocelectomy was followed. However, no improvement in the semen parameters was observed. Subsequently, microsurgical testicular sperm extraction was performed on the male patient in an attempt to retrieve sperm from testicular tissues, but the outcomes were negative. In response to these findings, the patient was advised to undergo intramuscular injections of human chorionic gonadotropin (hCG) at a dosage of 3,000 IU on alternate days, in conjunction with the daily administration of clomiphene citrate at 30 mg. Improvement in sperm parameters was seen after six months, leading to the successful intracytoplasmic sperm injection and the development of six blastocysts. The use of hCG significantly improved the semen quality, and frozen embryo transfer resulted in clinical pregnancy after endometrial preparation, highlighting the utilization of hCG therapy in varicocele cases for enhanced sperm retrieval and pregnancy success.

## Introduction

Varicocele is a condition in which the veins inside the scrotum become enlarged. The incidence of varicocele in infertile couples is about 17-41%, but 4-30% in the general population [1]. A physical examination of the testes should be performed in both upright and resting positions. It feels like a sack of worms when touched with hands. Only large varicoceles, which are easy to feel with the hands, are linked with infertility. Treatment of varicocele is needed when the male partner of the couple attempting to conceive has a varicocele and has met all the conditions of varicocele, and the female partner has a normal fertility evaluation or a possible treatable cause of infertility [2].

Management of varicocele, which is a treatable cause of male infertility, might improve the success rate of sperm retrieval. It is believed that varicocele repair increases the semen quality in men with infertility [3]. Varicocele disturbs spermatogenesis and results in an increased sperm DNA fragmentation index; it also decreases sperm motility, count, and normal morphology. Sperm DNA fragmentation decreases the outcome of in vitro fertilization (IVF), natural conception, and intrauterine insemination. Reparation of varicocele by surgical procedure significantly increases sperm quality in around 60% of males with infertility [4].

For the treatment of varicocele, microsurgical varicocelectomy has achieved a global application; it reserves the testicular artery and lymphatic vessels, ligates all the spermatic basal veins, and lowers the chances of complications. In male patients with infertility, it recovers the semen factors and also boosts the rate of pregnancies in female partners [5]. It also benefits patients with severe oligoasthenospermia and nonobstructive azoospermia by improving their semen factors. For treating varicocele, microscopic varicocelectomy has become the ‘‘golden standard’’ treatment method [5].

The use of human chorionic gonadotropin (hCG) has given the best results in males who underwent varicocele repair, but the repair did not improve the outcome. hCG also improved testosterone production in the testis in males having problems with Leydig cells [6]. In males, luteinizing hormone (LH) is produced by the part of the brain called the anterior pituitary in response to a hormone called gonadotropic-releasing hormone, which is secreted by the hypothalamus. It helps the Leydig cells in the testicles make more testosterone. In males with the condition called hypogonadotropic hypogonadism (HH) or males who use testosterone externally, the lack of LH causes a significant drop in testosterone within the testis. Spermatogenesis is disturbed without intratesticular testosterone, and by using hCG to replace lost LH, spermatogenesis can be restored. It helps in the restoration of spermatogenesis by restoring the level of testosterone [7]. This case report is a representation of a 32-year-old male patient suffering from varicocele and its treatment using hCG.

## Case presentation

Patient information

The couple, who had experienced four years of primary infertility, visited the assisted reproductive technology clinic situated in the rural area of the Wardha region. The male patient’s age was 32 years, and the female patient’s age was 29 years. The male patient was diagnosed with varicocele, while all test reports for the female partner were found to be normal.

Medical and surgical examination

The couple had no past surgical history, but the male was advised about surgical intervention and surgery for varicocele as part of the IVF treatment plan. After three months post-surgery, the patient underwent examination again, revealing no improvement. In the physical examination, the basal metabolic index (BMI) of both partners was observed to be in the normal range, with the husband having a BMI of 23 kg/m^2^ and the wife having a BMI of 21 kg/m^2^.

Couple investigation

The husband’s semen analysis was conducted to assess various parameters, including sperm count and motility. The analysis revealed severe azoospermia, indicating the absence of sperm in the semen. Follicle-stimulating hormone (FSH) and LH levels were examined and found to be in the lower range. Additional hormonal assessments, including testosterone, showed lower levels. A scrotal ultrasound was performed to evaluate the function, size, and severity of the varicocele, distinguish between subclinical and clinical varicocele, and assess any testicular abnormalities. FSH levels were checked and found to be within the normal range, while anti-Müllerian hormone levels were found to be in a good range (2.8 ng/mL). The uterine examination, conducted through ultrasound and hysteroscopy, aimed to rule out structural abnormalities, with a focus on assessing endometrial thickness. All factors were found to be normal, indicating no abnormalities in the female reproductive system. This implies that the female partner did not exhibit any factors contributing to infertility.

Diagnosis

The couple’s difficulty in achieving pregnancy for the first time signifies a case of primary infertility. The male patient was diagnosed with obstructive azoospermia and was also experiencing varicocele.

Treatment

**Figure 1:**
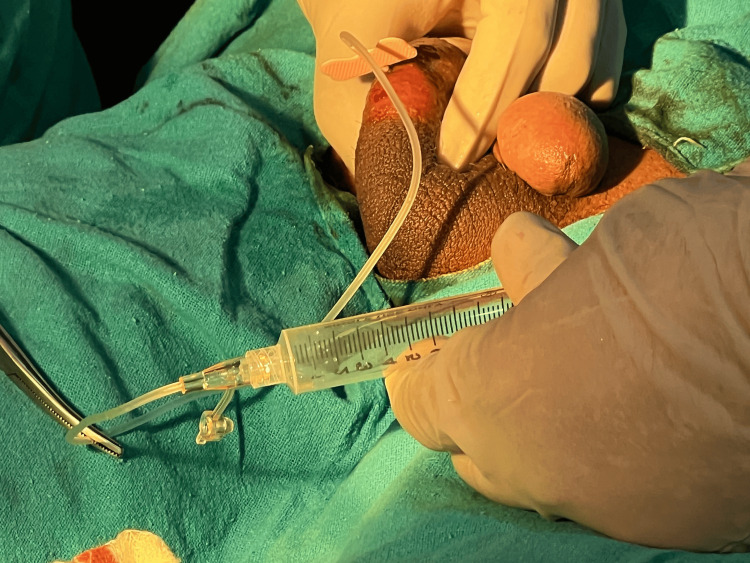
Extraction of sperm by TESA procedure TESA, testicular sperm aspiration

The patient underwent consultation and was recommended a microsurgical varicocelectomy to address varicocele as part of our infertility treatment plan, to which the patient consented. Subsequently, the patient underwent the surgical procedure for varicocele repair. Three months post-surgery, semen analysis was conducted, revealing no significant improvement in sperm quality and motility. Consequently, the patient was advised to undergo testicular sperm aspiration (TESA), and after consenting, the procedure was scheduled and carried out. However, TESA did not yield sufficient tissue, leading to the decision to perform microdissection testicular sperm extraction (micro-TESE). micro-TESE involves a minor scrotal incision for the extraction of testicular tubules, which are then meticulously examined for sperm. Despite micro-TESE, no sperm were identified in the testicular tissue sample. Figure 1 shows the image of the extraction procedure for sperm via TESA.

In light of the issue, the male patient underwent a blood test, which revealed below-normal levels of FSH and LH. Following consultation, the patient was prescribed intramuscular injections of hCG (3,000 IU on alternate days) along with clomiphene citrate (30 mg daily) for a six-month duration. Semen analysis was performed every two months during this period. After six months, improvements were observed in semen parameters, with a 20% increase in sperm motility. These motile sperms were utilized for the intracytoplasmic sperm injection (ICSI) procedure.

Concurrently, the female partner underwent ovarian stimulation for ovum pickup (OPU). During the OPU procedure, seven high-quality metaphase II oocytes were retrieved. The limited viable sperm obtained were then used for fertilization, resulting in the cultivation of six average-quality blastocysts by day 5. The patient was recommended for frozen embryo transfer (FET), and after consenting, the six embryos were cryopreserved on two separate straws. After a month, an embryo transfer (ET) was performed, and the patient was instructed to rest at the center for a few hours before being discharged.

Follow-up

After 14 days of ET, the female partner was called to the center and advised to check her β-hCG level. When the report was studied, it was seen that the β-hCG level was 231 mIU/mL, which indicates successful implantation of the embryo and a positive sign of pregnancy. Then, the doctors prescribed some medicines to the female partner and gave some instructions to follow. The female partner was then supervised for nine months, and on the due date, she delivered a healthy baby girl.

## Discussion

This case underscores the complexities associated with male infertility and the challenges encountered in its management and treatment. It illuminates the inherent difficulties in addressing male infertility effectively. Despite the corrective intervention of varicocele through surgical means, the resultant outcome was not favorable, necessitating advanced procedures such as testicular biopsy and TESA.

Under normal scrotal conditions, a homeostatic countercurrent heat exchange mechanism operates between the efflux of the pampiniform plexus and the inflow of testicular arterial blood, maintaining a cooler scrotal temperature [8]. However, varicocele modifies this physiological process, leading to an elevation in scrotal temperature. The heightened temperature adversely affects Leydig cell functionality, resulting in reduced testosterone production. Increased temperature acts as an inhibitor of C-17,20-lyase, impacting the conversion of 17-OH-progesterone to androstenedione and subsequently to testosterone. This inhibition contributes to decreased androgen levels and an increase in 17-OH-progesterone levels [8].

In the context of male HH, testosterone therapy proves effective in facilitating the maturation and maintenance of secondary sex characteristics [8]. However, for inducing spermatogenesis, the administration of gonadotropins is essential. In cases where pulsatile gonadotropin-releasing hormone is contraindicated or undesirable, hCG serves as an alternative source of LH activity, stimulating testosterone secretion by Leydig cells [9].

Lower testosterone levels impede the activity of epididymal 5-α-reductase, decreasing the conversion to dihydrotestosterone (DHT). Conversely, insufficient conversion to DHT results in an increase in aromatization to estradiol. Some authors propose the use of aromatase inhibitors in cases of male infertility characterized by low testosterone levels and a compromised testosterone-to-estradiol (T:E) ratio. Others corroborate the observation of reduced testosterone and T:E ratio in men with varicocele, suggesting significant improvement following varicocelectomy [8].

Kamal et al. investigated the outcome of microsurgical varicocelectomy on sperm quality and pregnancy outcome, finding significant improvement in sperm quality and motility [8]. Similarly, in a study by Li et al., surgical varicocelectomy was shown to improve semen parameters and pregnancy outcomes in men with palpable varicocele [10]. A study by Morini et al. indicated that surgical correction of varicocele significantly decreases sperm issues, suggesting potential benefits in related live cases [11]. This formed the basis for our decision to perform a varicocelectomy on the patient; however, the results were not satisfactory.

Maintaining optimal estrogen levels is crucial, as they adversely influence testicular function by suppressing the activity of Leydig cells’ 17β-hydroxysteroid dehydrogenase (17β-HSD). This inhibition impedes the conversion of androstenedione to testosterone, leading to reduced testosterone synthesis. In a study by Kamal et al., an elevated androstenedione/testosterone ratio was observed following hCG administration before varicocelectomy, with subsequent normalization of the ratio observed three months post-surgery [8]. This observation suggests compromised 17β-HSD activity in males with varicocele.

In a study by Pan et al., hCG significantly improved sperm production in hypogonadism azoospermic males [12]. Similarly, after varicocelectomy, hCG administration improved sperm parameters and pregnancy rates. In a study by Vicari et al., hCG alone increased sperm production in males with isolated HH [13]. Therefore, we recommended hCG treatment for the patient post-varicocelectomy, aligning with previous research findings. Overall, this case report emphasizes the effectiveness of administering hCG to address infertility concerns in patients with varicocele.

## Conclusions

In conclusion, this case underscores the challenges associated with treating male infertility linked to varicocele. Despite the conventional varicocelectomy and the unsuccessful micro-TESE, the devised approach involving hCG therapy and clomiphene citrate demonstrates significant improvement in sperm parameters after a six-month period. Subsequently, the utilization of motile sperm for ICSI, in conjunction with oocytes retrieved from the female partner, yielded six average-quality blastocysts (3AB and 3BB). Successful cryopreservation and FET resulted in a positive clinical pregnancy, highlighting the efficacy of hCG therapy in enhancing sperm quality and ultimately achieving a successful pregnancy outcome. Ongoing supervision and potential additional interventions may prove crucial to ensure a healthy and sustained pregnancy.
